# Implementation of the computer tomography parallel algorithms with the incomplete set of data

**DOI:** 10.7717/peerj-cs.339

**Published:** 2021-02-24

**Authors:** Mariusz Pleszczyński

**Affiliations:** Faculty of Applied Mathematics, Silesian Technical University of Gliwice, Gliwice, Śląskie, Poland

**Keywords:** Computer tomography, Parallel algorithms, Incomplete set of data, Big Data, Signal and data processing

## Abstract

Computer tomography has a wide field of applicability; however, most of its applications assume that the data, obtained from the scans of the examined object, satisfy the expectations regarding their amount and quality. Unfortunately, sometimes such expected data cannot be achieved. Then we deal with the incomplete set of data. In the paper we consider an unusual case of such situation, which may occur when the access to the examined object is difficult. The previous research, conducted by the author, showed that the CT algorithms can be used successfully in this case as well, but the time of reconstruction is problematic. One of possibilities to reduce the time of reconstruction consists in executing the parallel calculations. In the analyzed approach the system of linear equations is divided into blocks, such that each block is operated by a different thread. Such investigations were performed only theoretically till now. In the current paper the usefulness of the parallel-block approach, proposed by the author, is examined. The conducted research has shown that also for an incomplete data set in the analyzed algorithm it is possible to select optimal values of the reconstruction parameters. We can also obtain (for a given number of pixels) a reconstruction with a given maximum error. The paper indicates the differences between the classical and the examined problem of CT. The obtained results confirm that the real implementation of the parallel algorithm is also convergent, which means it is useful.

## Introduction

Computer tomography has a very wide field of applicability. Except the classical application in medicine (Donegani et al., 2020), for which the concepts and the first tomograph have been developed (Hounsfield, 1972), the methods of computer tomography are used in many other areas as well. In general, the computer tomography founds an application whenever there appears a need of examining the object inside, without affecting its structure (Cozzolino et al., 2020; Gong, Nie & Xu, 2020; Yao, Liu & Xu, 2020; Kamath et al., 2016).

The whole process of tomograph examination can be divided into two main stages: collection of data and transformation of these data into image. The research executed for the first stage consists in selecting the radiation beam, the radiation type, or in minimization of the necessary radiation dose, which is especially important for medical tomography (Malczewski, 2020). The second stage assumes that the data are already collected and ready to be transformed into the image of interior of the investigated object. This part is executed with the aid of the selected reconstruction algorithms. Such algorithms can be divided into two groups: analytical algorithms (Averbuch & Shkolnisky, 2003; Lewitt, 1983; Waldén, 2000) and algebraic algorithms (Andersen, 1989; Gordon, Bender & Herman, 1970; Guan & Gordon, 1996). The former group of algorithms is very good in the classical applications of computer tomography, when the collected data satisfy the assumption of Kotelnikov Theorem (Natterer, 1986), which states, in general, that the initial data must be sufficiently numerous and of sufficiently good quality. However sometimes this assumption is impossible to fulfill, because, for example, of the size of examined object or its inaccessibility. We have then the situation of incomplete data set.

As the studies have shown, the application of analytical algorithms is ineffective in the case of incomplete data set, whereas the algebraic algorithms can be successfully used (Hetmaniok, Ludew & Pleszczyński, 2017).

The approach in case of the incomplete set of data is important with respect of possible applications, for example, in examination of the mine coal seam (see “Problem with the Incomplete Set of Data”). The seam of coal may include the accumulations of unwanted rocks (which is important for economic reasons) or the areas of compressed gas (which is even more important because of the safety reasons). The compressed gas may explode during the coal seam exploitation causing the rock and gas ejections and the bumps, extremely dangerous for life and health of working people. For example, in Polish coal mines, in the second half of the 20th century 31 miners died because of that reason. The biggest catastrophe of this kind happened on the 7th September 1976 in the coal mine in Nowa Ruda, where 19 miners have been killed. Obviously there exist some methods of examining the mine coal seam before its exploitation, but these methods are invasive, time- and energy-consuming, which means that they are not very safe and significantly increase the mining costs.

The research, conducted so far, shows that the selected algorithms, belonging to the group of algebraic algorithms, can be applied for solving the problem with the incomplete set of data (for example in examination of the mine coal seam), however the specifics of such received data causes that the reconstruction process takes significantly more time than in classical approach. Therefore, the algorithms using the parallel computations are proposed, the aim of which is to reduce the time needed to examine the internal structure of the studied object. Till now such algorithms have been developed and studied only theoretically (Hetmaniok, Ludew & Pleszczyński, 2017), and simulated only for the one-thread process. Goal of the current paper is to investigate the convergence of such algorithms in case when the parallel computations are executed with the real application of several cores/threads. This study will be the ground for the further research on effectiveness of these algorithms in comparison with the sequential algorithms.

### Ideas of the computer tomography

Assuming that the distribution of density in the interior of examined object is described by means of function *f*(*x*,*y*) (which can be the continuous function as well as the discrete function), the computer tomography is meant to reconstruct this function on the basis of scans of this object along some paths *L*. Each scan (projection) informs how many energy is lost by the given ray along the given path. Since every sort of materia is characterized by the individual absorption of energy (e.g., for X-rays, where it refers to water which is assigned material density 998.2 (kg/m^3^), attenuation coefficient 0 Hounsfield units (HU), muscle tissue has 1,060 (kg/m^3^) and 41 HU, blood: 1,060 (kg/m^3^) and 53 HU, bone males: 3,880 (kg/m^3^) and 1,086 HU), therefore the function *f*(*x*,*y*) can be retrieved on the ground of such data, described by equation $p = {p_L} \equiv \ln{{{I_0}} \over I} = \int_L f(x,y)dL$, where *L* denotes the mention path, *p*_*L*_ means the projection obtained in this path, *I*_0_ is the initial intensity of radiation, whereas *I* is the final intensity. At the beginning of the last century it has been proven (Radon, 1917) that the reconstruction of function *f*(*x*,*y*) is possible based on the infinitely many projections. Assumption of possessing infinitely many projections is impossible to fulfill in real life. In practice it is possible to get only finite number of projections, therefore very important for development of computer tomography are the works (Louis, 1984a, 1984b) presenting the conditions connecting the number of projections (this number consists of the number of scanning angles and the number of rays in one beam) with the possibility of reconstructing the function *f*(*x*,*y*). The analytical algorithms, mentioned above, are based, among others, on the Fourier and Radon transforms, and also on the concept of filtered backprojection, therefore they cannot be used for the projections of insufficiently good quality. For this reason we will consider only the algebraic algorithms.

### Algebraic algorithms

In the algebraic algorithms it is assumed that the examined object is located in the rectangle (or square, very often), which can be divided into *N* = *n*^2^ smaller congruent squares (pixels). Size of these pixels enables to assume that the reconstructed function *f*(*x*,*y*) takes on unknown constant value in each pixel. Thanks to this, by knowing the initial energy of radiation and the energy of the given ray after passing through the investigated object (in this way we know the value of lost energy, that is the value of projection), the equation of path passed by the ray, the density of discretization (number of pixels) and keeping in mind the fact that every sort of materia is characterized by the individual capacity of energy absorption, we can calculate the total energy loss for the given ray (the value of projection) as the sum of energy losses in every pixel met by the ray. The loss of energy in one pixel is proportional to the road passed by the ray in this pixel (this value is known) and to the unknown value of function *f*(*x*,*y*) in this pixel (values of this function should be found). Considering every ray individually we obtain a system of linear equations (single scanning is represented by one line of this system):

(1) $$AX = B,$$

where *A* denotes a matrix of dimension *m* × *N* containing the non-negative real elements, *m* means the number of projections and *N* = *n*^2^ means the number of pixels, *X* denotes a matrix (vector) of length *N* containing the unknown elements—each *i*th element of this matrix represents the unknown constant value of the reconstructed function *f*(*x*,*y*) for *i*th pixel, finally *B* is a matrix (vector of length *m*) of projections.

The algebraic algorithms differ from each other in the method of solving the system (1). Since matrix *A* has some specific characteristics:

- it is a sparse and nonsymmetric matrix (the vast majority of elements is equal to zero^1^ 1It is easy to notice that a single row of length *n*2 has at most 2*n* − 1 nonzero elements),
- it is not a square matrix (mostly there is definitely more rows than columns),
- it is nonsingular matrix,
- its dimension is very big,

therefore for solving the system (1) we cannot apply the classical methods dedicated to the solution of the systems of linear equations.

#### Kaczmarz algorithm

**Figure 1:**
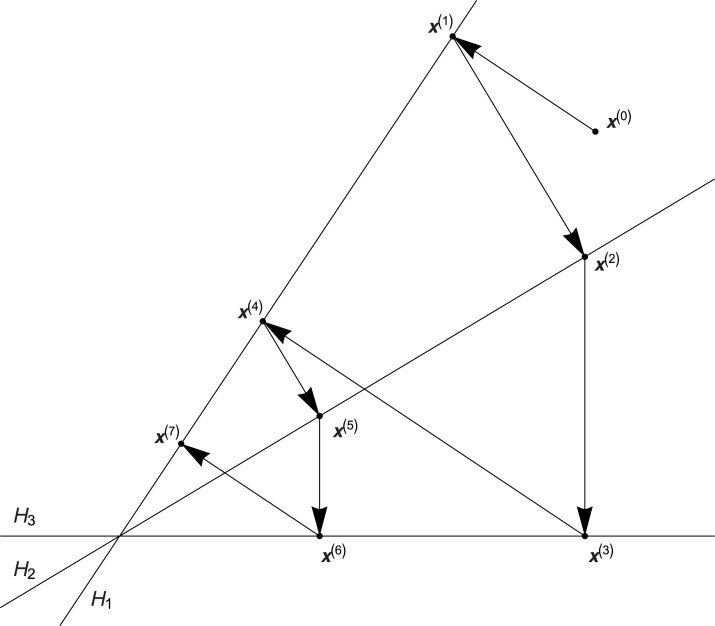
Geometric interpretation of the Kaczmarz algorithm.

Most of the algebraic algorithms is based on the Kaczmarz algorithm, the convergence of which has been proven at first for the square systems (Kaczmarz, 1937) and next also for the rectangle systems (Tanabe, 1971). This algorithm starts by selecting any initial solution ${{\bf x}^{(0)}} \in {{\mathbb{R}}^N}$, and every successive approximation of solution **x**^(*k*)^, $k \in {\mathbb{N}}$, is computed as the orthogonal projection of the previous solution onto hyperplane *H*_*i*_, *i* = (*k* − 1,*m*) + 1, where hyperplane *H*_*i*_ is represented by the respective line of system (1), that is ${H_i} = \{ {\bf x} \in {{\mathbb{R}}^N}:{{\bf a}^i} \circ X = {p_i}\}$, where operation ∘ is defined as the classic scalar product of vectors from space ${{\mathbb{R}}^N}$, **a**^*i*^ denotes the *i*th row of matrix *A*, *p*_*i*_ means the *i*th projection, that is *p*_*i*_ = *b*_*i*_ is the *i*th element of vector *B*, *i* = (*k* − 1, *m*) + 1. Geometric interpretation of this algorithm is presented in Fig. 1.

#### Algorithm ART

For many years the Kaczmarz algorithm was not applied. Thanks to papers (Gordon, Bender & Herman, 1970; Tanabe, 1971) it was modified to algorithm ART and in this form it has found an application in computer tomography. Algorithm ART, similarly like the Kaczmarz algorithm, starts by choosing any initial solution ${{\bf x}^{(0)}} \in {{\mathbb{R}}^N}$ and the next approximations of solution are created by means of the following relation

(2) $${{\bf x}^{(k + 1)}} = {{\bf x}^{(k)}} + {\lambda _k}\displaystyle{{{p_i} - {{\bf a}^i} \circ {{\bf x}^{(k)}}} \over {{\rm \parallel }{{\bf a}^i}{{\rm \parallel }^2}}}{{\bf a}^i}$$

where the same notations hold, as before, additionally ||·|| means the norm of vector (that is the length of vector) and $\lambda \in {\mathbb{R}}$ denotes the relaxation coefficient.

Similarly like in case of other algorithms discussed in this paper, we can speed up the convergence of ART algorithm by using the physical property of the studied phenomenon—each sort of matter is characterized by the specific capacity of absorbing the energy of penetrating radiation. Value describing this capacity is nonnegative, therefore after determining the solution **x**^(*k*^ ^+ 1)^ we operate with the aid of operator *C*, called in literature the constraining operator, taking in this paper the following form

(3) $$C:{{\mathbb{R}}^N} \to {{\mathbb{R}}^N},\;\;C({x_1},{x_2}, \ldots ,{x_N}) = ({y_1},{y_2}, \ldots ,{y_N}),$$

where

(4) $${y_i} = \left\{ {\matrix{ {{x_i},} & {{x_i} \ge 0,} \cr {0,} & {{x_i} < 0,} \cr } } \right.\;1 \le i \le N$$

Several years after introducing the ART algorithm, it has been proven in paper (Trummer, 1984) that this algorithm is convergent if 0 < λ_*k*_ = λ < 2, whereas in case when λ does not have to be constant, the ART algorithm is convergent if 0 < liminf λ_*k*_ ≤ limsup λ_*k*_ < 2. In specific case when λ_*k*_ = λ = 1 the ART algorithm is the Kaczmarz algorithm.

Obviously there exist many other algebraic algorithms, like for example the algorithms ART-3 (Herman, 1975), MART (Gordon, Bender & Herman, 1970; Verhoeven, 1993), SIRT (Gilbert, 1972), SART (Andersen & Kak, 1984) and others. We focus in the current paper on the ART algorithm (and its parallel adaptations) because, as the research shows, the other algorithms are characterized by the same convergence rate and the algorithm ART is simple in implementation which is its great advantage.

The algebraic algorithms, adapted to the problem with incomplete set of data, appeared to be convergent, stable and detecting the non-transparent element, which means that they are very useful (Hetmaniok, Ludew & Pleszczyński, 2017). However the main disadvantage of these algorithms is their convergence speed—less than 20 iterations is required for solving the problem with complete set of data^2^ 2One iteration means the consideration of all lines in system (1), that is the execution of *m* (1) projections., whereas few hundred, or even more than one thousand, iterations is often needed for solving the problem with incomplete set of data. The stopping condition for the ART algorithm (as well as for the other algorithms discussed in this article) can be defined as the assumed number of executed iterations or the assumed precision of the obtained approximate solution. System of algebraic linear equations similar to considered one are overdetermined ad as a rule are inconsistent. The Kaczmarz method and its modifications converge to some “pseudosolution”. The research concerning this subject were also performed—one can notice that the successive approximate solutions, after reaching some level of precision, “circulate” around the theoretical exact solution, however they are contained within some *N*-dimensional sphere with central point located in this exact solution and radius proportional to the level of error burdening the input data.

One of possibilities to defeat the described disadvantage consists in applying the parallel calculations in the process of determining the solution of system (1). Among many algorithms using this approach we select the parallel block algorithms.

### Parallel block algorithms

**Figure 2:**
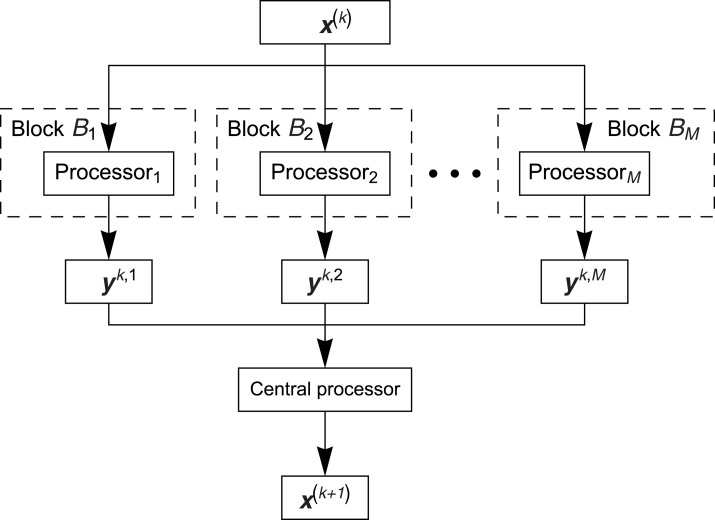
Scheme of the parallel block algorithm.

Parallel block algorithms are created to use the parallel work of many processors/threads, however the number of threads is relatively small (differently like in case of using the threads of graphics cards with CUDA technology). The general concept of these algorithms consists in partition of the system of Eq. (1) into blocks (the number of blocks corresponds to the number of used threads) so that the set of indices of rows of matrix *A* is presented in the form of sum: {1,2,…,*m*} = *B*_1_∪ *B*_2_∪…∪ *B*_*M*_, where, in the standard approach, we have *B*_*i*_ ∩ *B*_*j*_ = Ø for *i* ≠ *j*, and the cardinalities of sets *B*_*i*_, 1 ≤ *i* ≤ *M*, are equal more or less (very often the blocks are formed so that the first block includes about ${m \over M}$ first rows, the second block includes ${m \over M}$ next rows, and so on). The blocks work parallel in such a way that they have the same initial vector (starting solution) **x**^(*k*)^, *k* ≥ 0, in every block the algorithm (for example the ART algorithm) is performed on the rows of matrix *A* belonging to this block, next, after executing one iteration (by using all available rows), the approximate solution **y**^(*k*,*i*)^, *k* ≥ 0, 1 ≤ *i* ≤ *M*, from each block is returned. After that the solutions are averaged and such average solution **x**^(*k*^ ^+ 1)^ can serve as the initial solution for all blocks in the next iteration. Graphical illustration of the parallel block algorithm is presented in Fig. 2.

**Figure 3:**
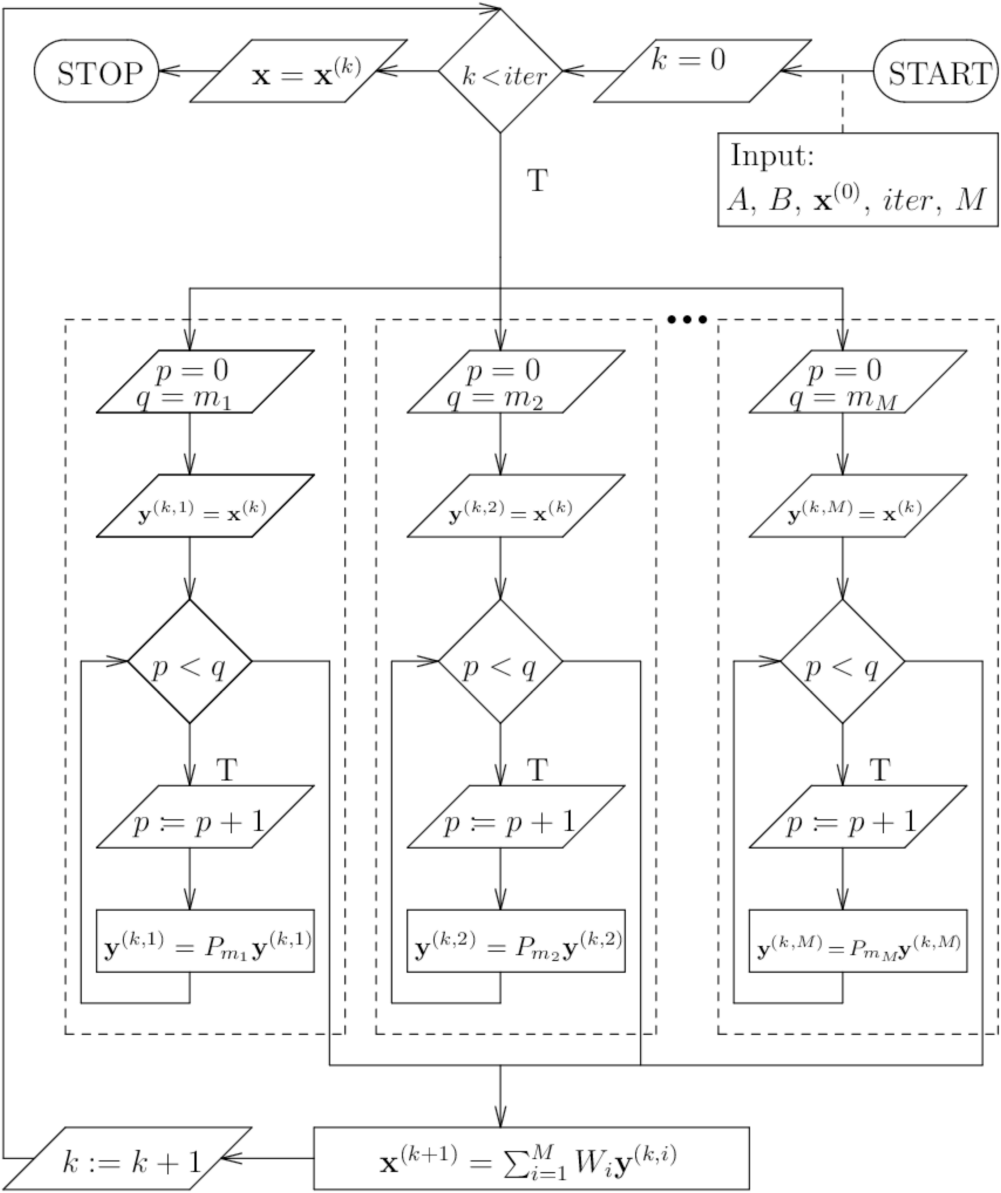
Scheme of the parallel block algorithm.

The Fig. 3 shows the BP algorithm in more detail (block algorithm).

Assuming the partition of matrix *A* into blocks (vector *B* of projections is partitioned in the same way), the parallel block algorithm PB (introduced by the Author) can be presented in the following way: we select the initial solution **x**^(0)^, we calculate the solution **x**^(*k*^ ^+ 1)^, *k* ≥ 0, according to formula

(5) $${{\bf x}^{(k + 1)}} = \sum\limits_{i = 1}^M {W_i}{{\bf y}^{(k + 1,i)}}$$

where

(6) $${{\bf y}^{(k + 1,i)}} = {Q_i}{{\bf x}^{(k)}}$$

whereas *Q*_*i*_ denotes the operator composing the operators *P*, that is

(7) $${Q_i} = {P_{i,b1}}{P_{i,b2}} \ldots {P_{i,bs}}$$

where operator *P*_*i*,*bj*_ means the execution of projection (2), defined in the ART algorithm, onto the *j*th hyperplane of block *B*_*i*_ possessing *s* elements. Component *W*_*i*_, occurring in formula (5), is responsible for averaging the solutions obtained from the respective blocks and is defined by the square matrix of dimension *N* having the following form

$$W_i=diag \{w_1^i,w_2^i,\ldots,w_N^i\}$$

where

$$w_j^i=\frac{\sum\limits_{q\in B_i}a_{q,j}}{\sum\limits_{q=1}^Ma_{q,j}}$$

and *a*_*q*,*j*_ denotes the element of matrix *A* located in *q*th row and *j*th column, 1 ≤ *i* ≤ *M*, 1 ≤ *j* ≤ *N*.

### Problem with the incomplete set of data

Problem of the incomplete set of data in the classical form is discussed, for example, in Andersen (1989). The incomplete set of data is presented there, and also in many other publications, in the following way: for the parallel beam there is executed 100 directions of scanning (for angles of range between 0 and 180 degrees) and in each beam there are 121 rays (then the set of data is complete). The scans at angles between 56 and 80 degrees are missing in this set (which generates the incomplete set of data—about 14% of data is missing in this set, about 86% of data is given there). However in the considered case we are very far from this situation—the scans are performed only between two opposite walls, which means that the scanning angles are included within the right angle, so one can say that we have 50% of data in our disposal (this estimation is still too optimistic). The Author analyzed such situation and came to the conclusion that the interior of the examined object can be reconstructed also in such conditions (convergence, stability, influence of noises, occurrence of non-transparent elements were investigated), but this reconstruction takes quite a long time. Therefore the solutions, different than the ones used in various classical approaches, were sought and are still required (the Author tested them in his cited paper), like: selection (on the way of appropriate investigations) of optimal values of parameters, other sorts of algorithms, sorting the rows in matrix of the system of equations, introduction of chaotic and asynchronous algorithms, introduction of parallel algorithms (parallel-block and block-parallel). Separate application of these approaches (or of their combinations) caused, the most often, the improvement of the convergence speed, however this improvement was not big enough to accept it as sufficient. Similar studies were also carried out in other studies (e.g., Jiang & Wang (2003), most often assuming a complete set of data—therefore this case requires separate studies). For example, Sørensen & Hansen (2014) shows that the row sorting effect of *A* does not significantly improve time. Many authors have studied block and parallel algorithms (also block parallel algorithms), including graphs, many other approaches were also used (a large part of them was also investigated by the author for this issue) (Censor et al., 2008; Drummond et al., 2015; Elfving & Nikazad, 2009; Gordon & Gordon, 2005; Sørensen & Hansen, 2014; Torun, Manguoglu & Aykanat, 2018; Zhang & Sameh, 2018).

**Figure 4:**
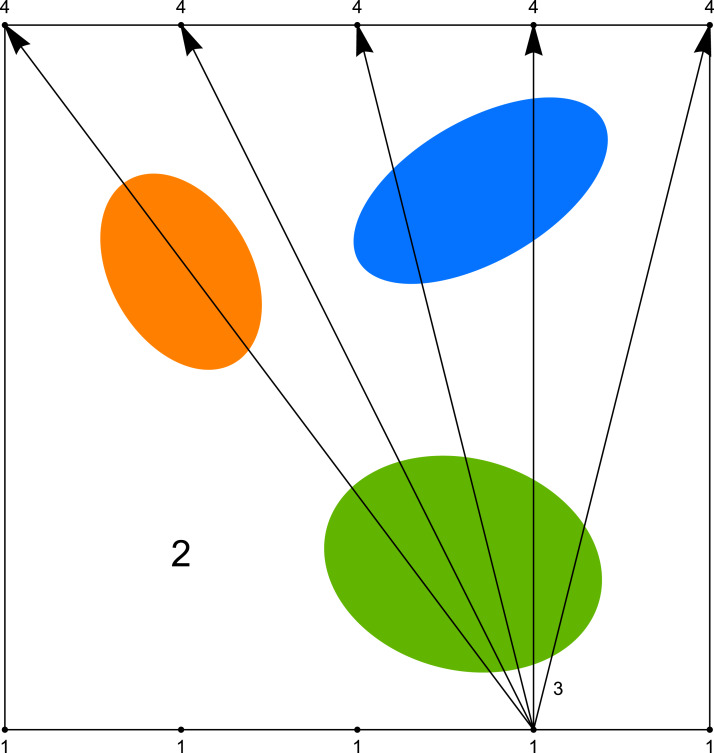
System (1 × 1) where 1—sources of rays, 2—object under research, 3—rays, 4—detectors.

**Figure 5:**
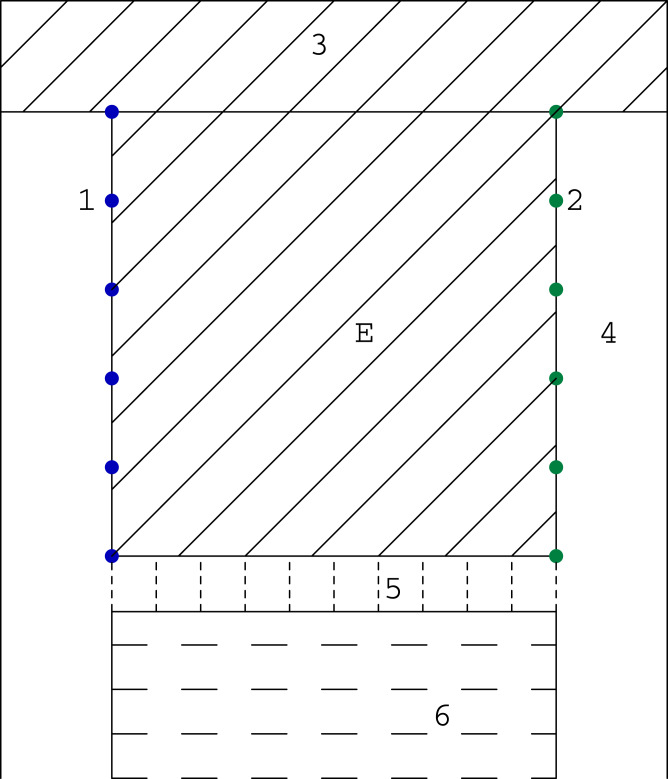
The scheme of a coal bed working: 1—sources; 2—detectors; 3—unmined coal; 4—heading; 5—longwall with mechanized lining, belt conveyor flight, heading machine so on; 6—caving or filling; E—researching coal bed.

As we mentioned before, the computer tomography, considered in its classical sense, requires the projection of a very good quality (many scanning angles, many rays in one beam). However in study of some problems, like for example in examination of the mine coal seam, the size of investigated object or difficult access to it does not allow to get such type of projection. Then we have the problem with the incomplete set of data. The most often we can use the data obtained only between two opposite walls of the studied object (such system will be called the (1 × 1) system), and sometimes between pairs of two opposite walls (such system will be called the (1 × 1, 1 × 1) system). System (1 × 1) is presented in Fig. 4 and an example of its application (coal seam testing) is shown in the Fig. 5.

### Mathematical models of phantoms

In the seam of coal, in which we search for dangerous areas of compressed gas or unwanted interlayers of barren rocks, the distribution of density is discrete and the densities of included elements differ from each other. Therefore the models used for testing the convergence of discussed algorithms possess the discrete distribution of high contrast. Thus, the two-dimensional function describing the distribution of density takes the following form

(8) $$f(x,y) = \left\{{\matrix{ {{c_1},} & {(x,y) \in {D_1} \subset E,} \cr {{c_1},} & {(x,y) \in {D_2} \subset E,} \cr \cdots & {} \cr {{c_s},} & {(x,y) \in {D_s} \subset E,} \cr } } \right.$$

where ${c_i} \in {\mathbb{R}}$, 1 ≤ *i ≤ s*, *E* = {(*x*,*y*) − 1 ≤ *x*, *y* ≤ 1}, and the regions *D*_*i*_, 1 ≤ *i* ≤ *s*, are disjoint (more precisely, the area if their common part is equal to zero).

An additional parameter, affecting the convergence of investigated algorithm PB, is the number of sources and detectors. This parameter is denoted by *pkt* and influences directly the dimension of matrix *A* of system (1)—it determines the number of rows in this matrix. We assume in this article that the number of sources and detectors are equal and we reject (for uniqueness—we eliminate the case when the ray runs along the mesh discretizing the square *E*) the first and the last projection. Then we have *m* = *pkt*^2^ − 2.

### Results of experiments

To show that the PB algorithm is convergent in practise (not only in theory) one has to prove that it is possible to select the optimal^3^ 3As the research shows, the exact optimal values are impossible to determine, because they depend on various elements, like the expected value of parameter l, form of function *f* (*x*, *y*), the system of data collection and so on. Whereas it is possible to observe some approximate relation between these values. values of parameters *m* and λ for the given resolution (number of pixels *n*^2^ = *N*), similarly like in case of the sequential algorithms. Obviously, for such selected values of reconstruction parameters it should be possible to retrieve the sought function with the given precision.

Table 1 presents the times needed to achieve the error Δ < 0.05 in reconstruction of function *f*_1_(*x*,*y*) for *n* = 40 with the use of 3 threads, depending on the values of parameters *m* and λ, whilst

(9) $$\Delta = \mathop {\max }\limits_{1 \le i \le N} \left| {{f_1}(pi{k_i}) - {{\tilde f}_1}(pi{k_i})} \right|$$

where *pik*_*i*_ is the *i*th pixel, *f*_1_ denotes the exact values of sought function, whereas ${\tilde f_1}$ describes its reconstructed values.

**Table 1:** Dependence of the reconstruction time [s] for the given *n* on the values of parameters *m* and λ (value > 10 means the time longer than 10 s).

*λ* ↓ *m* →	32	34	38	40	42	44	48	50	52	54
0.25	>10	>10	>10	>10	>10	>10	3.963	4.321	4.336	3.9
0.5	>10	>10	>10	2.683	2.403	2.465	1.856	1.997	1.997	1.794
0.75	2.356	>10	3.229	1.7	1.45	1.544	1.154	1.248	1.202	1.107
1	1.732	2.87	2.434	1.17	0.967	1.092	0.796	0.889	0.827	0.78
1.25	1.326	2.137	1.95	0.904	0.78	0.827	0.608	0.64	0.608	0.609
1.5	1.232	1.825	1.701	0.78	0.687	0.702	0.577	0.484	0.593	0.624
1.75	1.155	1.638	1.56	0.733	0.608	0.609	0.639	0.562	0.717	0.764
2	1.223	1.7	1.716	0.702	0.608	0.842	0.874	0.749	1.107	1.061
2.5	1.903	3.307	>10	>10	>10	>10	>10	>10	>10	>10
***λ* ↓ *m* →**	**58**	**60**	**62**	**64**	**68**	**70**	**72**	**74**	**78**	**80**
0.25	3.963	4.18	4.243	4.15	3.744	3.978	3.885	3.572	3.697	3.978
0.5	1.919	1.935	1.919	1.935	1.747	1.81	1.872	1.747	1.794	1.81
0.75	1.232	1.186	1.17	1.17	1.154	1.342	1.154	1.201	1.233	1.264
1	0.92	0.795	0.796	0.827	0.921	1.061	0.952	0.967	0.983	1.076
1.25	0.78	0.671	0.671	0.67	0.811	1.014	0.858	0.92	0.936	1.076
1.5	0.811	0.64	0.655	0.655	0.858	1.077	0.921	0.951	0.936	1.154
1.75	1.045	0.749	0.905	0.858	1.061	1.342	1.108	1.154	1.201	1.357
2	1.716	1.201	1.451	1.482	1.404	1.716	1.514	1.575	1.778	1.794

Error (9) takes this form only in the initial stages of algorithm usability testing. We then refer to an exact solution, which we do not know of course in real cases. However, such an approach has an undoubted advantage—by conducting a series of experiments, we can estimate the number of iterations (for given values of the reconstruction parameters) to obtain a given quality of reconstruction.

The obtained results are like expected. The shortest time is for *m* = 50 and λ = 1.5 (more detailed investigation around this value of λ, with step 0.1, showed that this is the best result in this case). Calculations executed for other resolutions and other functions describing the distribution of density gave similar results concerning the proportion of *n* to *m* and to the value of λ.

**Figure 6:**
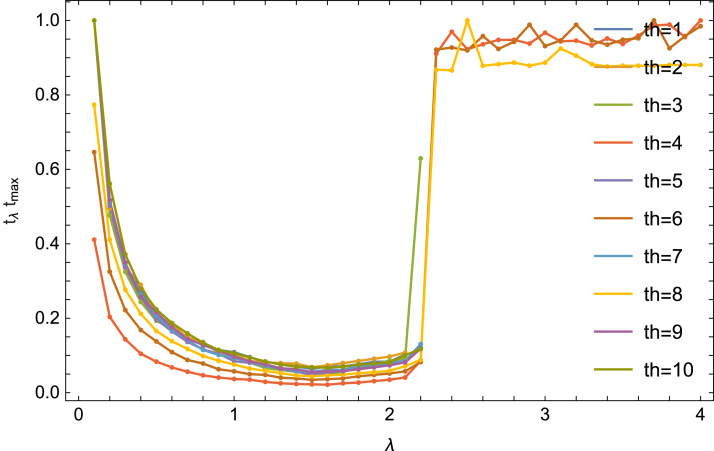
Dependence of the reconstruction on λ parameter value for an equal number of threads.

The literature (see e.g., Gordon & Gordon, 2005) shows that in the case of a complete set of data, the selection of the optimal λ parameter depends heavily on the number of threads. The Fig. 6 shows the reconstruction time (quotient of the time *t*_λ_ for obtaining the error (9) Δ < 0.01 for a given value of λ and for a given number of threads and the time *t*_max_ maximum for this number of threads) from the number of threads *th*.

In the case of a incomplete set of data, the situation is different. For any number of threads, the optimal value for λ is similar. The results for the number of threads 4, 6 and 8 are also interesting: the algorithm converges there for 0.1 ≤ λ ≤ 10 (the Fig. 6 it is shown for λ ≤ 4).

Table 2 presents optimal values of the λ parameter for the step *s* = 0.1 and for the step *s* = 0.01.

**Table 2:** Optimal values of the λ parameter for the step *s* = 0.1 and for the step *s* = 0.01.

*s* ↓ *th* →	1	2	3	4	5	6	7	8	9	10
0.1	1.5	1.5	1.6	1.6	1.5	1.5	1.5	1.5	1.5	1.6
0.01	1.41	1.41	1.56	1.55	1.53	1.44	1.50	1.50	1.56	1.62

The research was carried out for many different functions of the density distribution and for many values of the reconstruction parameters selected for these functions. We now present graphically the obtained results for two selected examples. The first presented function of the density distribution will be the function *f*_1_, which according to the formula (8) takes the form

$$f_1(x,y)= \left\{{\matrix{1, & (x,y)\in [-0.7,-0.4]\times[-0.5,0.2]\cr 2,& (x,y)\in [-0.2,0.2]\times[-0.1,0.1]\cr 3, & (x,y)\in [-0.2,0.2]\times[0.3,0.5]\cr 4, & (x,y)\in [0.4,0.7]\times[0.4,0.7]\cr 0, & \text{otherwise}}} \right.$$

**Figure 7:**
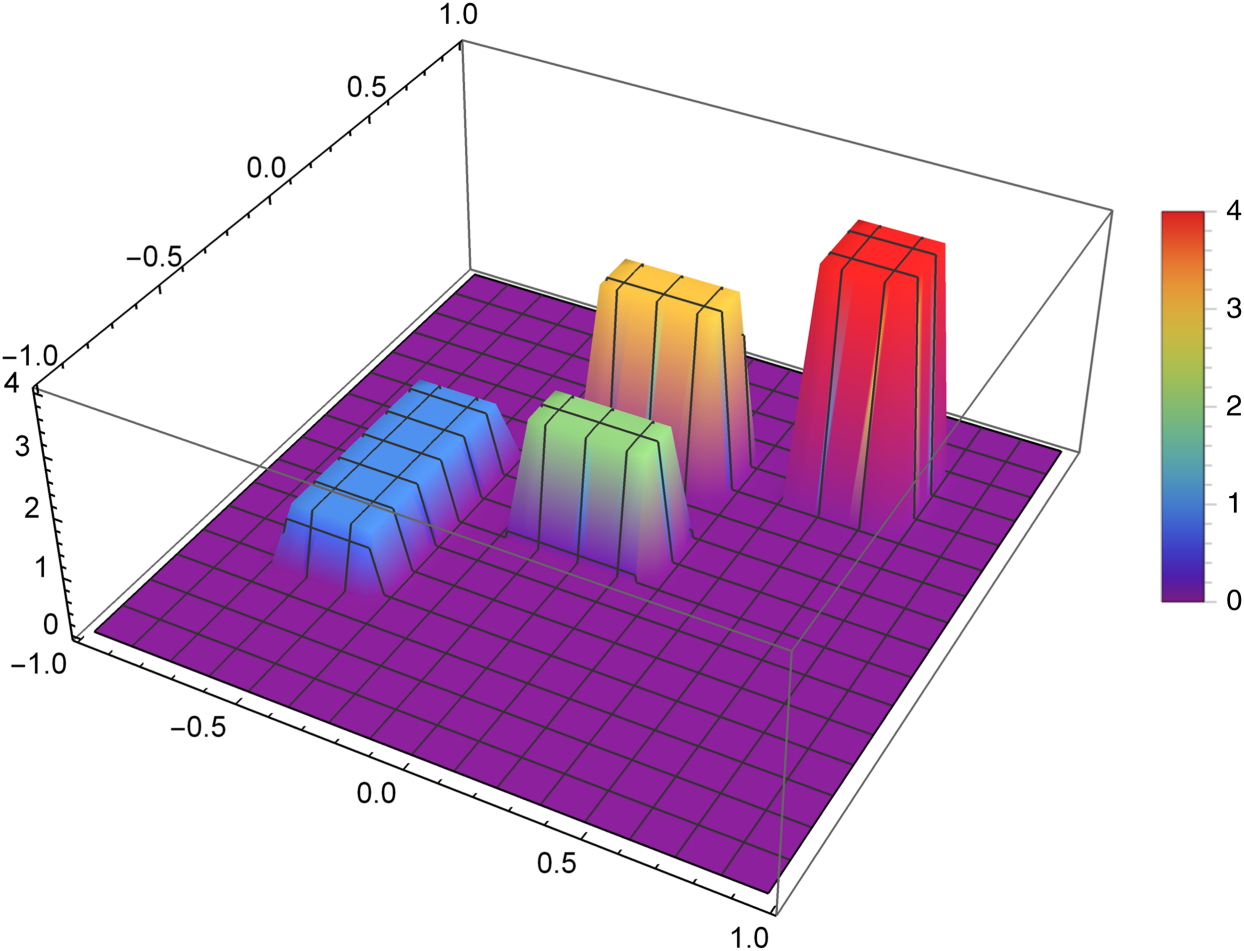
Reconstructed function *f*_1_(*x,y*) for *n* = 40, *m* = 50, λ = 1.5 and 3 threads.

**Figure 8:**
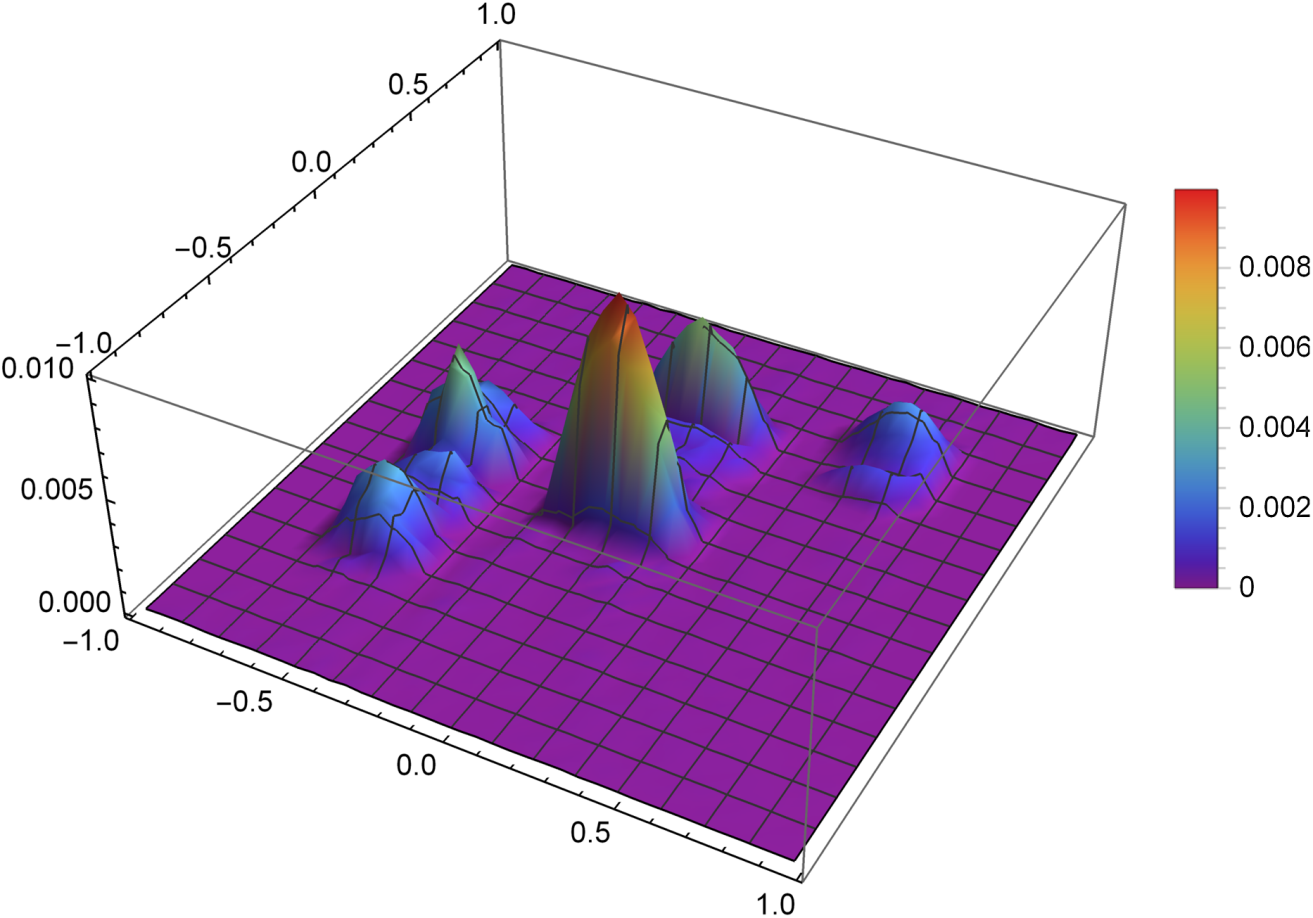
The error Δ of the reconstruction of the function *f*_1_(*x,y*) for *n* = 40, *m* = 50, λ = 1.5 and 3 threads.

Figure 7 presents the reconstruction of function *f*_1_ for *n* = 40, *m* = 50, λ = 1.5 and 3 threads. Figure 8 presents the error Δ (see (9)) of this reconstruction.

The second presented function of the density distribution will be the function *f*_2_, which according to the formula (8) takes the form

$$f_2(x,y)= \left\{{\matrix{1, & (x,y)\in [0,0.5]\times[0.3,0.5] \cr 2, & (x,y)\in [-0.4,0]\times[-0.6,0.7] \cr 3, & (x,y)\in [-0.6,-0.4]\times[-0.1,0.1] \cr 4, & (x,y)\in [0,0.3]\times[-0.5,-0.3] \cr 0, & \text{otherwise}}} \right.$$

**Figure 9:**
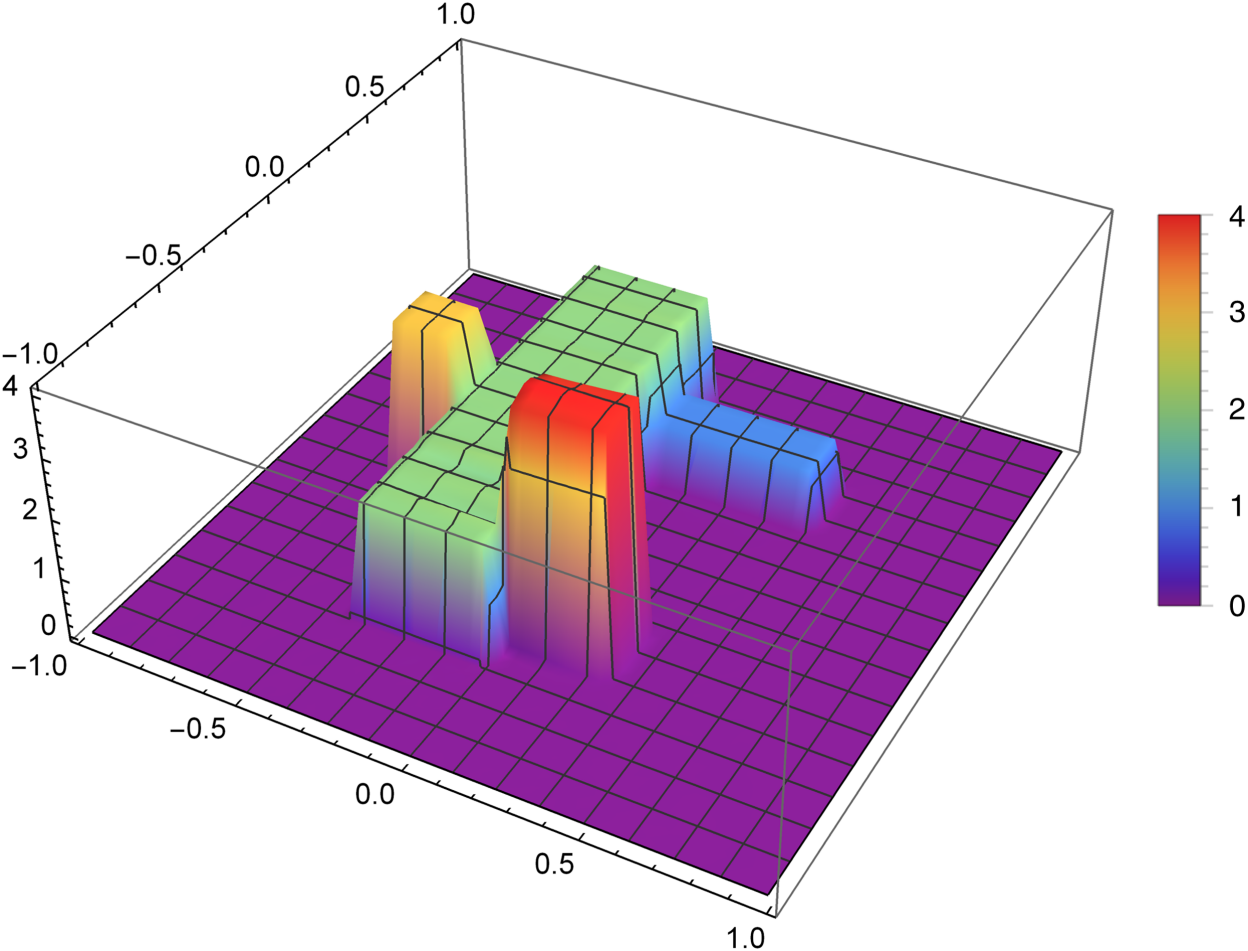
Reconstructed function *f*_2_(*x,y*) for *n* = 60, *m* = 80, λ = 1.5 and 8 threads.

**Figure 10:**
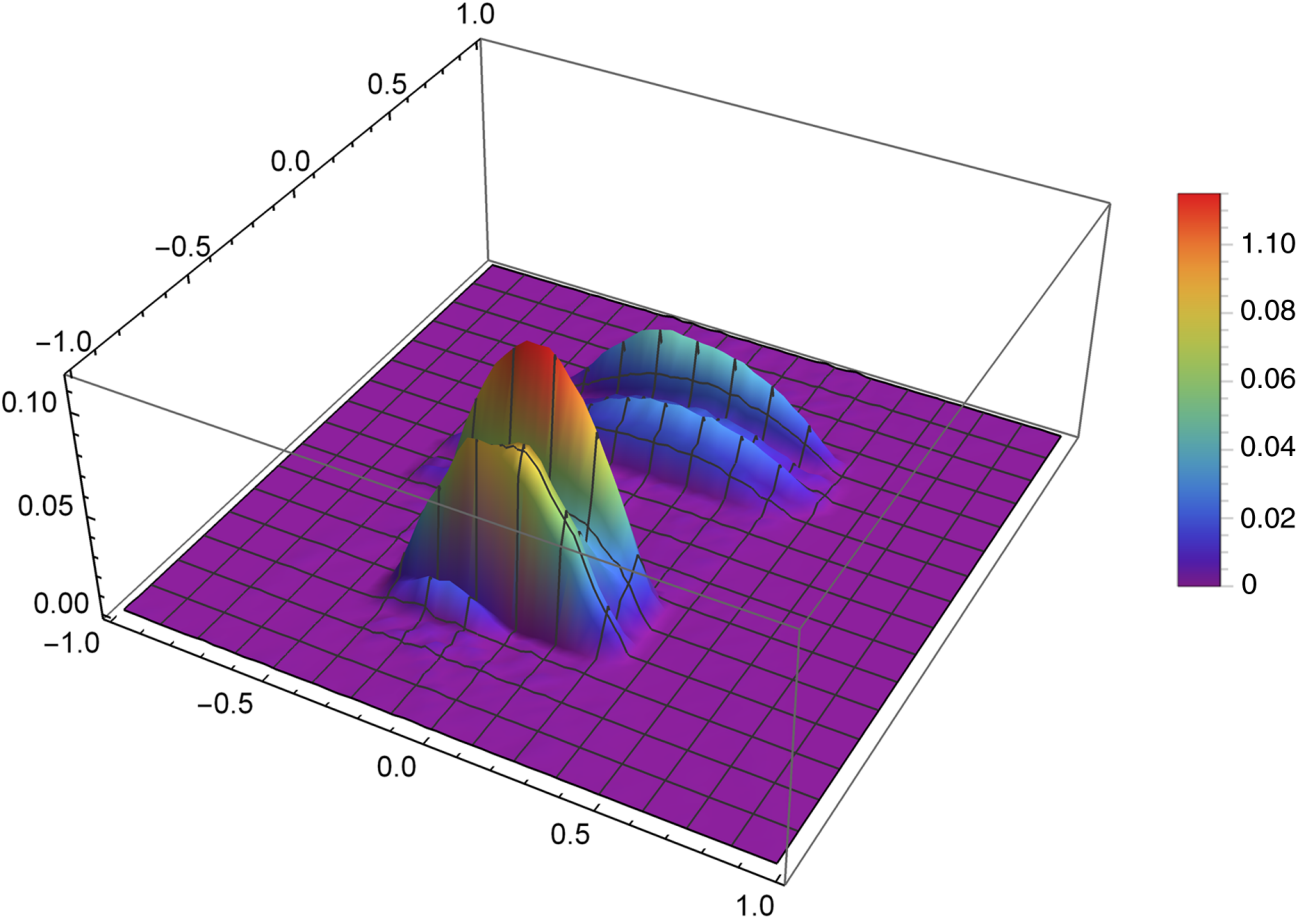
The error Δ of the reconstruction of the function *f*_2_(*x,y*) for *n* = 60, *m* = 80, λ = 1.5 and 8 threads.

Figure 9 presents the reconstruction of function *f*_2_ for *n* = 60, *m* = 80, λ = 1.5 and 8 threads. Figure 10 presents the error Δ (see (9)) of this reconstruction.

**Figure 11:**
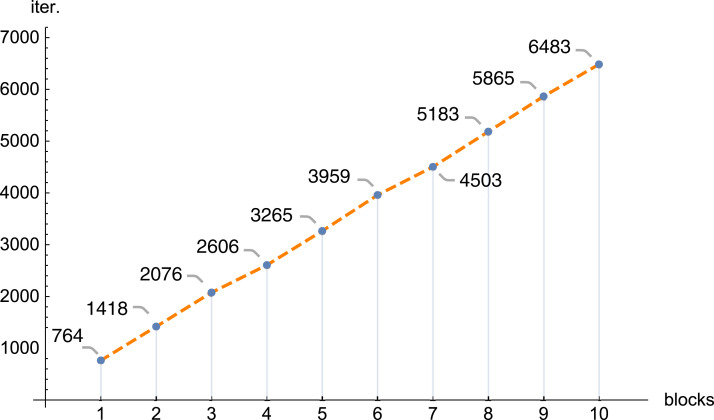
The number of iterations (iter.) needed to get the error Δ < 0.1 depending on the number of blocks, *n* = 100, *m* = 150, λ = 1, *f*_1_(*x,y*).

**Figure 12:**
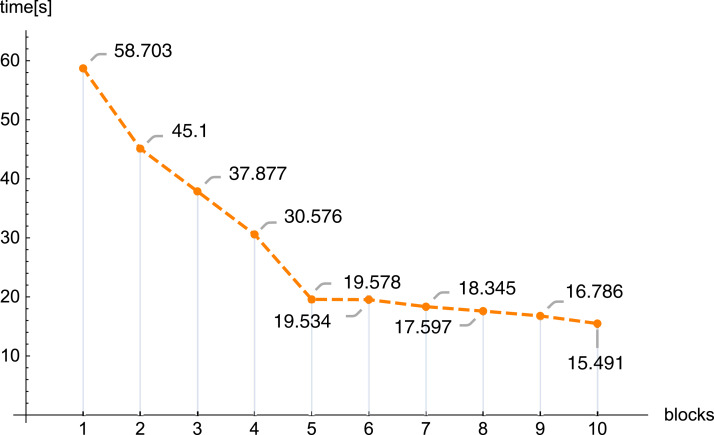
Execution time 1,000 iterations (iter.) depending on the number of blocks, *n* = 100, *m* = 150, λ = 1, *f*_1_(*x,y*).

In the next figures (Figs. 11 and 12) we demonstrate, for selected examples, the correctness of behavior of the reconstruction parameters, that is, more precisely, the number of iterations required to obtain the given error Δ depending on the number of blocks, together with the time needed to execute 1,000 iterations depending on the number of blocks.

Computer programs, realizing the presented research, were written in the following languages: *C*# from Visual Studio 2017 and Mathematica 12. The study was conducted with the aid of computer with Windows 7 Professional system, equipped with 16 GB RAM, processor Intel Core i7 3.2 GHz (12 threads). It is also worth to mention that the largest considered systems of equations possessed 160,000 unknown elements (*n* = 400) and were composed from 359,998 equations (*m* = 600) and the data concerning the coefficients of such systems used more that 6 GB of disk space.

## Conclusions

The conducted investigations, presented in this article, show that the introduced PB algorithm is useful, also in practice. For the assumed resolution one can select, with big precision, the values of other reconstruction parameters, in order to minimize the required calculations, however, the selection of optimal values of the reproduction parameters is of a different nature than in the classical task. Dividing matrix *A* of equation system (1) into blocks, the information about the examined object is poorer in each block (in comparison with information delivered by the full matrix *A*), therefore the number of iterations increases with the number of blocks. Research has shown that this increase is linear. If we refer to the number of iterations for one thread, then as the number of blocks increases, the number of iterations increases, and the increase is approximately 0.835 the number of iterations for one thread (this is shown in Fig. 7, but for other cases it is similar). The application of bigger number of threads reduces significantly the time needed to execute the iterations. In the initial phase, increasing the number of threads reduces the execution time of 1,000 iterations. On average, the execution time for 1,000 iterations on *n* threads is approximately 76.39% of the execution time for 1,000 iterations on *n* − 1 threads. Then (from 6 threads) this percentage drops significantly (the reason is a much smaller amount of information that individual blocks have).

In future there are planned the further tests for optimizing the reconstruction parameters in order to develop the biggest possible advantage of PB algorithm, and other algorithms using the parallel calculations, over the sequential algorithm. The current paper gives the basis for this planned research.

## Supplemental Information

10.7717/peerj-cs.339/supp-1 Supplemental Information 1 Computer Code. The Mathematica file creates (using parallel computations) matrices of a system of linear equations. The C # files solve (using parallel computations) this system of equations.

## Additional Information and Declarations

### Data Availability

The following information was supplied regarding data availability: The program codes are available in the Supplemental Files.
